# Emergency department visits of older adults within 30 days of discharge: analysis from the pharmacotherapy perspective

**DOI:** 10.31744/einstein_journal/2020AO4871

**Published:** 2019-10-17

**Authors:** Fabiana Silvestre dos Santos, Bianca Menezes Dias, Adriano Max Moreira Reis

**Affiliations:** 1 Universidade Federal de Minas Gerais, Belo Horizonte, MG, Brazil.

**Keywords:** Emergency service, hospital, Aged, Drug therapy

## Abstract

**Objective:**

To analyze, from the pharmacotherapy perspective, the factors associated to visits of older adults to the emergency department within 30 days after discharge.

**Methods:**

A cross-sectional study carried out in a general public hospital with older adults. Emergency department visit was defined as the stay of the older adult in this service for up to 24 hours. The complexity of drug therapy was determined using the Medication Regimen Complexity Index. Potentially inappropriate drugs for use in older adults were classified according to the American Geriatric Society/Beers criteria of 2015. The outcome investigated was the frequency of visits to the emergency department within 30 days of discharge. Multivariate logistic regression was performed to identify the factors associated with the emergency department visit.

**Results:**

A total of 255 elderly in the study, and 67 (26.3%) visited emergency department within 30 days of discharge. Polypharmacy and potentially inappropriate medications for older adults did not present a statistically significant association. The diagnosis of heart failure and Medication Regimen Complexity Index >16.5 were positively associated with emergency department visits (OR=2.3; 95%CI: 1.04-4.94; p=0.048; and OR=2.1; 95%CI: 1.11-4.02; p=0.011), respectively. Furthermore, the diagnosis of *diabetes mellitus* and chronic kidney disease were protection factors for the outcome (OR=0.4; 95%CI: 0.20-0.73; p=0.004; and OR=0.3; 95%CI: 0.13-0.86; p=0.023).

**Conclusion:**

The diagnosis of heart failure and Medication Regimen Complexity Index >16.5 were positively associated with the occurrence of an emergency department visit within 30 days of discharge.

## INTRODUCTION

The increased number of the elderly in society determines a greater use of healthcare services, including emergency departments.^1^ A visit of the elderly to the emergency department is often a sentinel event, since it contributes towards the decline of health status, and thus is associated with an elevated risk of negative results, such as revisits to the emergency departments, hospitalization, and functional decline.^2^ The multimorbidity, use of polypharmacy, Complexity of the pharmacotherapy, and fragility are among the factors associated with these negative results in health.^2 , 3^

The return to emergency departments after hospital discharge often reflects a transition of care held in an inadequate manner. Despite clinical importance and relevance for the organization of the healthcare systems, after hospital discharge, the standard of use of emergency services is not well understood and investigated, which contributes to underestimation of the extent of its use.^4^

Approaches centered only on hospital readmission provide an incomplete understanding of acute care after hospital discharge, making it difficult to identify the factors that contribute towards the need for subsequent use of the healthcare system, especially the health problems managed in emergency services.^4 , 5^

The factors related to age, functionality, polypharmacy, complexity of pharmacotherapy, inappropriate medications for older adults, and multimorbidity can be associated with return visits to emergency services after hospital discharge.^2 , 3^ Additionally, the use of emergency services after hospital discharge may be considered a predictor of the fact that the process of transition of care is ineffective. This event may cause more overcrowding and increased costs in healthcare services.^1 , 4 - 6^

## OBJECTIVE

To analyze, from the perspective of pharmacotherapy, the factors associated with visits of elderly individuals to the emergency departments within 30 days of hospital discharge.

## METHODS

### Study design and setting

This is a cross-sectional study with the elderly carried out in a public hospital of the Southeastern region of Brazil, which provides care to civil servants. The age of ≥60 years was adopted for the definition of “elderly,” as per established by the World Health Organization for developing countries.^7^

### Sample

The minimum inclusion of 246 elderly subjects in the study was estimated, considering the following premises: finite population, 20% prevalence of visits of the elderly to urgency services after hospital discharge,^8^ 95% confidence interval (95%CI), 5% margin of error, and test statistics of a population. Sample calculation was made using Open Epi software, version 2.0 *.*

### Selection criteria

The study included elderly patients hospitalized by the units of internal medicine and geriatrics, during the period from April to November 2017. The exclusion criteria were defined as patients who died during the index admission, were in the index admission hospital for more than 60 days, or evaded the hospital, returned electively, or lost contact after discharge. The first admission during the period of the study was chosen as the index admission.^9^

### Ethical considerations

The study was approved by the Research Ethics Committee of the *Universidade Federal de Minas Gerais* , under opinion no. 1.952.130 and CAAE: 63612216.7.0000.5149. All patients and/or caregivers consented to participate by means of the Informed Consent Form.

### Data collection

The patients admitted to hospital for a period of more than 24 hours were invited to participate in the research, and this was considered their index admission. The identification of patients was done by a computerized report of the admission system of the hospital investigated. The following procedures were done: initial interview, data collection from the patient’s electronic medical records, and two telephone follow-ups after discharge (the first to confirm the medications prescribed upon discharge, and the second, to check the occurrence of the event). The first telephone contact was made within 72 hours after discharge and the second, within 30 days of discharge.

The outcome was the search for emergency departments within 30 days of discharge of the index admission, both at the hospital investigated and at other healthcare services. The visit to emergency departments was defined as a stay of the elderly patient for less than 24 hours, following national,^10^ and international^11^ parameters that establish time of observation in an emergency service. At each interview, sociodemographic functionality data were collected by exploring the patient’s medical records as to clinical characterizations and pharmacotherapy of the elderly patient. The diagnoses of admission and readmissions were classified as per the International Classification of Diseases, tenth edition (ICD 10). Comorbidities were evaluated using the Charlson comorbidity index (CCI).^12^ The potentially inappropriate medications for older adults were described according to the American Geriatric Society/Beers criteria of 2015.^13^ The complexity of the pharmacotherapy was calculated using the complexity of pharmacotherapy index,^14^ the version translated into Brazilian Portuguese of the Medication Regimen Complexity Index (MRCI).^15^ The complexity of pharmacotherapy was stratified as high complexity, yes (MRCI >16.5), or no (MRCI ≤16.5), according to the MRCI standardization proposed for the elderly in Brazil.^16^ Vulnerability was assessed by the instrument Vulnerable Elders Survey (VES-13), transculturally adapted to Brazil.^17^ The diagnoses and medications of risk for readmission were identified using the definition by Taha et al.^9^

### Statistical analysis

Determination of frequency for the dichotomous variables was done with a descriptive analysis, and the numerical variables were described as mean±standard deviation (SD) or median (interquartile range - IQR). The Shapiro Wilk test was used for normality analysis. The numerical variables were dichotomized by the median. The association between the independent variables and visit to the emergency department within 30 days was conducted as per Student’s *t* test, Mann-Whitney’s test, chi-squared test, and Fisher’s exact test, observing the premises of each test. The odds ratio (OR) was calculated with a 95% confidence interval. The level of statistical significance was set at p<0.05. The variables that presented an association with the emergency department visit with p<0.25 were selected for multivariate logistic regression. The forward stepwise method was used to obtain the final model, with the p<0.05 variables remaining. Adaptation of the final model was evaluated by the Hosmer-Lemeshow test, considering adequate the p value >0.05.

## RESULTS

**Figure 1:**
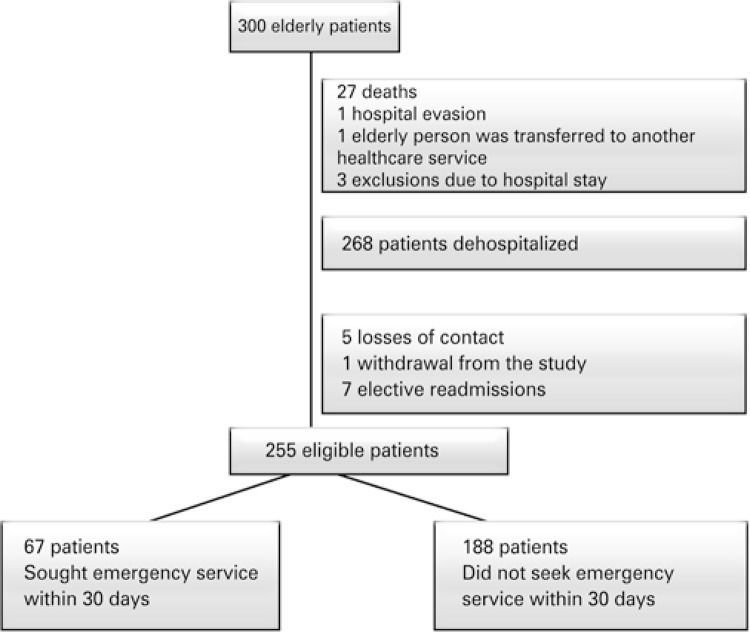
Study flowchart

Three hundred participants were recruited for the study, and of these, 27 (9%) died during hospitalization, one (0.3%) elderly man evaded the hospital, one (0.3%) elderly person was transferred to another healthcare service, and three (1%) patients were excluded due to prolonged hospital stay. Of the 268 patients who were discharged from hospital, there were five (1.7%) losses to follow-up, one (0.3%) withdrawal from the study, and seven (2.3%) exclusions due to elective rehospitalization. In this way, 255 participants were eligible for undergoing analyses, and of these, 67 elderly (26.3%) needed to seek an emergency department within 30 days of hospital discharge ( Figure 1 ).

Among 67 elderly individuals who visited an emergency department within 30 days after the index admission, 35 (52.2%) remained in the department for less than 24 hours, and 32 (47.8%) were readmitted. The frequency of the emergency department visit 30 days after the index hospitalization was 26.3%, for readmission it was 12.5%, and of the elderly who remained in the department for less than 24 hours it was 13.7%.

The emergency department of the hospital investigated was sought by 54 (80.6%) of 67 elderly individuals who needed urgency or emergency care. The fast track and emergency rooms of hospitals in the Brazilian National Healthcare System (SUS – *Sistema* Único *de Saúde* ) were sought by 8 (12.9%) elderly, and 5 (7.5%) preferred care in private services or those of the health insurance companies. The sociodemographic, clinical, and pharmacotherapeutic characteristics of elderly persons are described on table 1 . Considering that in both groups the female sex and age ≥75 years were more frequent, as to sex and age there was no statistically significant difference between the elderly who did or did not visit the emergency department.

**Table 1:** Sociodemographic, clinical and pharmacotherapeutic characteristics of the elderly

Characteristics	Visit to emergency services		Total n=255	p value

	Yes n=67	Noo n=188		
Sociodemographic				
Female	44 (65.7)	102 (54.3)	146 (57.3)	0.105
Age	77.0 (13.0)	75.0 (14.0)	75.0; (13.0)	0.470^*^
Clinical				
CCI	7.0 (6.0)	5.0 (2.0)	5.0 (2.0)	0.359^*^
VES-13	7.0 (6.0)	4.0 (5.0)	5.0 (6.0)	0.06^*^
Diagnosis of risk of readmission	52 (77.6)	145 (77.1)	197 (77.3)	0.541
HF	15 (22.4)	23 (12.2)	38 (14.9)	0.045
Cancer	11 (16.4)	18 (9.6)	29 (11.4)	0.130
COPD	10 (14.9)	24 (12.8)	34 (13.3)	0.655
Pneumonia	15 (22.4)	38 (20.2)	53 (20.8)	0.706
Diabetes	21 (31.3)	89 (47.3)	110 (43.1)	0.023
Stroke	7 (10.4)	38 (20.2)	45 (17.6)	0.332
Pharmacotherapy				
Medications prescribed at discharge				
ASA + clopidogrel	1 (2.9)	7 ( 3.7)	8 (3.1)	0.685^†^
Insulin	10 (14.9)	42 (22.3)	52 ( 20.4)	0.196
Opioid	13 (19.4)	19 (10.1)	32 (12.5)	0.049
Warfarin	6 (1.5)	8 (4.3)	14 (5.5)	0.207^†^
Enoxaparin	-	1 (0.5)	1 (0.4)	0.737^†^
Fondaparinux	-	-	-	-
Digoxin	-	-	-	-
PIME	122 (64.9)	43 (64.2)	165 (64,7)	0,913
Polypharmacy	49 (73.1)	125 (66.5)	174 (68,2)	0,316
Excessive polypharmacy	31 (16.5)	14 (20.9)	45 (17,6)	0,417
Medications of risk of readmission	28 (41.8)	65 (34.6)	93 (36,5)	0,292
Number of medications at discharge	6.0 (5.0)	6.0 (4.0)	6,0 (4,0)	0,210*
MRCI	12.0 (16.5)	16.5 (14.4)	17,0 (14,5)	0,167^*^

Results expressed as n (%) or median (interquartile range). * Fischer´s exact test; ^†^ Mann-Whitney test. CCI: Charlson comorbidity index; VES-13: Vulnerable Elders Survey; HF: heart failure; COPD: chronic obstructive pulmonary disease; ASA: acetyl salicylic acid; PIME: potentially inappropriate medications for older adults; MRCI: Medication Regimen Complexity Index.

Regarding the clinical characteristics, the statistically significant difference was evident between the groups (p=0.045), with greater frequency of heart failure in the aged group that visited an emergency department. The proportion of elderly persons with diabetes was significantly greater among those who did not seek an emergency department. As to functionality, there was a clear statistically significant difference (p=0.038), with greater values of VES13 in the elderly group that was seen at an emergency department. Considering the presence of a diagnosis of risk, there was no difference between the groups of elderly patients; we highlight the high prevalence of elderly persons with this characteristic in the sample studied.

Considering pharmacotherapy, the elderly who were discharged from the hospital with prescriptions of opioids sought the urgency service more than the individuals who did not receive a prescription for medications from this therapeutic group of drugs (p=0.049). The proportion of the elderly who use medications of risk for hospitalization showed no statistically significant difference, but it was more prevalent among the elderly who sought emergency care. The proportion of the elderly with high complexity pharmacotherapy showed no statistically significant difference among the groups, but was discretely more frequent among the elderly who were hospitalized.

In the univariate analysis, the visit to the emergency department showed an association with the female sex, heart failure, cancer, *diabetes mellitus* , chronic renal disease, asthma, atrial fibrillation, Vulnerable Elders Survey-13 (VES-13), high complexity pharmacotherapy, use of insulin, opioids, and warfarin. In the final multivariate design, the following variables remained, heart failure (OR= 2.3, 95%CI: 1.04-4.94), *diabetes mellitus* (OR=0.4; 95%CI: 0.20-0.73), chronic renal disease (OR=0.3; 95%CI: 0.13-0.9), and high complexity pharmacotherapy (OR=2.1; 95%CI: 1.11-4.02), as shown on table 2 .

**Table 2:** Univariate and multivariate analysis of factors associated to visits of the elderly to emergency department within 30 days of hospital discharge

Variable	Frequency of readmission		Univariate analysis		Multivariate analysis				
		
	Yes	No	Odds ratio (95%CI)	p value	Odds ratio (95%CI)		p value*		
Sociodemographic									
Sex									
Female	44 (65.7)	102 (54.3)	1.6 (0.90-2.88)	0.105	---		---		
Male	23 (34.3)	86 (45.7)	1						
Age (years)									
≥75	39 (58.2)	101 (53.7)	1.2 (0.68-2.19)	0.526	---		---		
<75	28 (41.8)	87 (46.3)	1						
Clinical									
Length of stay (days)									
≥12	40 (59.7)	101 (53.7)	1.3 (0.72-2.25)	0.398	---		---		
<12	27 (40.3)	87 (46.3)	1						
Diseases (ICD 10)									
Stroke (I64)									
Yes	11 (16.4)	26 (13.8)	1.2 (0.57-2.64)	0.606	---		---		
No	56 (83.6)	162 (86.2)	1						
Heart failure (I50)									
Yes	15 (22.4)	23 (12.2)	2.1 (1.01-4.26)	0.045	2.3 (1.04-4.94)		0.048		
No	52 (77.6)	165 (87.8)	1		1				
COPD (J44)									
Yes	10 (14.9)	24 (12.8)	1.2 (0.54-2.66)	0.655	---		---		
No	57 (85.1)	164 (87.2)	1						
Cancer (C00-C75)									
Yes	11 (16.4)	18 (9.6)	1.9 (0.83-4.16)	0.130	---		---		
No	56 (83.6)	170 (90.4)	1						
*Diabetes mellitus* (E10-E14)									
Yes	21 (31.3)	89 (47.3)	0.5 (0.28-0.92)	0.023	0.4 (0.20-0.73)		0.004		
No	46 (68.7)	99 (52.7)	1		1				
Pneumonia (J12-J18)									
Yes	15 (22.4)	38 (20.2)	1.1 (0.58-2.24)	0.706	---		---		
No	52 (77.6)	150 (79.8)	1						
Dementia (F00-F03)									
Yes	22 (32.8)	57 (30.3)	1.1 (0.62-2.05)	0.702	---		---		
No	45 (67.2)	131 (69.7)	1						
Chronic renal failure (N18)									
Yes	7 (10.4)	38 (20.2)	0.5 (0.20-1.09)	0.072	0.3 (0.13-0.86)		0.023		
No	60 (89.6)	150 (79.8)	1		1				
Asthma (J45)									
Yes	2 (3.0)	1 (0.5)	5.8 (0.51-64.51)	0.170^*^	---		---		
No	65 (97.0)	187 (99.5)	1						
Atrial fibrillation (I48)									
Yes	3 (4.5)	18 (9.6)	0.4 (0.13-1.55)	0.193	---		---		
No	64 (95.5)	170 (90.4)	1						
Hypertensive diseases (I10-I15)									
Yes	50 (74.6)	131 (69.7)	1.3 (0.68-2.41)	0.444	---		---		
No	17 (25.4)	57 (30.3)	1						
Palliative care									
Yes	8 (11.9)	14 (7.4)	1.7 (0.67-4.22)	0.261	---		---		
No	59 (88.1)	174 (92.6)	1						
Charlson comorbidity index									
≥5	39 (58.2)	104 (55.3)	1.1 (0.64-1.98)	0.682	---		---		
<5	28 (41.8)	84 (44.7)	1						
Functionality									
VES-13									
≥5	43 (64.2)	93 (49.5)	1.8 (1.03-3.26)	0.038	---		---		
<5	24 (35.8)	95 (50.5)	1						
Pharmacotherapeutic									
ASA + clopidogrel									
Yes	1 (1.5)	7 (3.7)	0.4 (0.05-3.25)	0.685^*^	---				---
No	66 (98.5)	181 (96.3)	1						
Insulin									
Yes	10 (14.9)	42 (22.3)	0.6 (0.29-1.30)	0.196	---				---
No	57 (85.1)	146 (77.7)	1						
Opioid									
Yes	13 (19.4)	19 (10.1)	2.1 (0.99-4.62)	0.049	---				---
No	54 (80.6)	169 (89.9)	1						
Warfarin									
Yes	6 (9.0)	8 (4.3)	2.2 (0.74-6.63)	0.207^*^	---			---	
No	61 (91.0)	180 (95.7)	1						
PIME									
Yes	43 (64.2)	122 (64.9)	0.7 (0.54-1.74)	0.916	---			---	
No	24 (35.8)	66 (35.1)	1						
Polypharmacy									
Yes	49 (73.10	125 (66.5)	1.4 (0.74-2.55)	0.316	---			---	
No	18 (26.9)	63 (33.5)	1						
High MRCI									
Yes	40 (59.7)	93 (49.5)	1.5 (0.86-2.67)	0.150	2.1 (1.11-4.02)			0.021	
No	27 (40.3)	95 (50.5)	1		1				

Results expressed as n (%). Hosmer-Lemeshow test: χ^2^=1.549. Degree of freedom = 5; p=0.907. * p values with variables calculated by Fisher´s exact test. ICD10: International Classification of Diseases, tenth edition; COPD: chronic obstructive pulmonary disease; VES-13. Vulnerable Elders Survey; ASA: acetyl salicylic acid; PIME: potentially inappropriate medications for older adults; MRCI: Medication Regimen Complexity Index.

## DISCUSSION

In the present study, about one fourth of the elderly people returned to emergency departments within 30 days of hospital discharge, and this visit had a positive association with heart failure and pharmacotherapy complexity, while *diabetes mellitus* and chronic renal disease were protective factors for the outcome. The unplanned return to emergency departments within 30 days of hospital discharge is rarely investigated, and the studies developed until now, alert to the importance of widening the investigations about this period, in order to increase knowledge as to the acute care based on hospitals of patients with recent discharge.^4 , 5 , 8 , 18^ Analysis of the care given during hospitalization, considering only hospital readmission, does not afford an integral view of the acute care given, and limits the development of actions in favor of improved transition of care, especially of the mature patients.

Rates of visits to emergency departments varied from 7.5 to 23.8 in prior studies,^4 , 5 , 8 , 18^ but these investigations did not exclusively cover the elderly and included surgical patients. No studies carried out in Brazil were identified. The frequency of emergency department visits (26.3%) is close to that of studies with greater frequencies. However, the comparison of studies is made difficult due to different methodologies, lack of uniformity in timeframes that characterize visits to emergency departments, and exclusion in some studies of the patients who visited the emergency service and were admitted to the hospital, as well as by the differences in emergency care organization in countries where the studies were conducted.

The return to emergency services related to heart failure is an important negative outcome, which also results in significant healthcare costs. The rate of return to emergency services by patients with heart failure was 20% in a Canadian study.^19^ In another investigation, patients with this diagnosis and aged ≥80 years were significantly more prone to returning, when compared to younger individuals.^20^ Still, in both studies, the use of antiarrhythmic medications was negatively associated with the occurrence of the outcome.^19 , 20^

Pharmacotherapy of patients with heart failure is complex and covers medications from various drug classes. Additionally, the multiple comorbidities that the elderly present increase even more the complexity of drug regimens. Complex drug regimens are associated with poor compliance with treatment,^21 , 22^ polypharmacy,^21 , 23^ and hospitalization due to adverse events.^24^ Acute decompensation of heart failure is an important determinant of search for emergency departments.^25 , 26^ Noncompliance due to pharmacotherapy complexity or the incorrect use of medications can contribute towards acute decompensation of heart failure.^27^ Elevated MRCI scores at hospital discharge provide a more general assessment of the risk associated with the complex drug regimen. Patients with elevated values of MRCI present with a greater probability of falling ill and show more negative results.^21 , 24^ These factors might explain the positive association between visits to emergency departments, heart failure, and high complexity of pharmacotherapy.

Absence of an association between pharmacotherapy complexity (MRCI >15, dichotomized by the mean) and emergency department visit within 30 days of hospital discharge was described for individuals aged over 50 years.^3^ The discrepancy relative to the association detected in our study may be attributed to the different profile of the population investigated, since the elderly present with a more complex pharmacotherapy. Additionally, the definition of elevated complexity (pharmacotherapy complexity MRCI >16.5) standardized for the elderly in Brazil^16^ was adopted, which allows a more appropriate classification.

The intensification of pharmacotherapy for the treatment of *diabetes mellitus* (measured by the increased dose of hypoglycemic medications, addition of other hypoglycemic agents, and addition of insulin) significantly decreased the risk of returning within 30 days to the urgency sector after discharge at a North-American hospital.^28^ It is possible to infer that, among the elderly individuals studied, there was intensification of pharmacotherapy, which contributed towards adequate control of the disease over the period of 30 days, explaining the inverse association identified in this study between having *diabetes mellitus* and visiting the emergency department within 30 days of hospital discharge. Nevertheless, it is worth pointing out that insulin, used by some diabetic patients, is a medication of risk, especially for the aged, which could contribute towards the search for healthcare services after hospital discharge.^9^ Nonetheless, from the point of view of pharmacotherapy, the lower search for emergency departments within 30 days by elderly people with chronic renal disease may also be attributed to optimization of treatment.

Investigation about the profile of and prevalence of search for healthcare services by individuals with diabetes showed a lower prevalence in patients with a longer period since the diagnosis, such as the elderly.^29^ The aged with diabetes can present with better self-care due to the greater knowledge of the disease and treatment, owing to the diagnosis made years before, and explaining the protective factor found for search for emergency services. Better care of one’s health, especially by women, explains the association with chronic renal disease. Women with chronic renal disease present with a lower rate of hospital readmission within 30 days. The greater frequency of women in the cases investigated may justify the association. However, it is important to point out that medications constitute an important determinant of hospital admission of individuals with chronic renal disease.^6^

The absence of an independent association between polypharmacy and return to the emergency department was described in a cohort of elderly Italians,^2^ which is in agreement with the findings of the present study. Nonetheless, for the elderly people of the same cohort, an independent association was found between excessive polypharmacy (use of ten or more medications, considering the previous 3 months) and visit to the emergency department 30 days after discharge.^2^ The authors point out that polypharmacy is a marker of multimorbidity, severity of the disease, fragility, and geriatric syndromes,^2^ conditions that can contribute towards negative results in health. It is important to consider polypharmacy as a parameter to guide actions in transition of care, seeking to avoid future adverse events in the elderly, since after 6-month follow-up, both polypharmacy and excessive polypharmacy showed an independent association with return to the emergency department.^2^

Our study presented with relevant information to guide actions seeking to optimize the transition of care of the elderly and reduce visits to the emergency department. Additionally, it is important to point out that this is a prospective investigation, in which telephone follow-ups were made after discharge, guaranteeing reliable data on the outcome. Another strength of the study is the determination of the association between the visit to an emergency department and the complexity of the pharmacotherapy utilized the exact definition of high complexity (MRCI >16.5) established in the standardization of MRCI for elderly Brazilians.^16^

Nevertheless, we present some limitations, such as its being carried out in a single hospital that provides care exclusively to civil servants, limiting generalization for patients seen at SUS hospitals or in private networks. Second, the study did not include elderly patients from surgical clinics, which could have underestimated the frequency of search for an emergency department. Finally, no visits to the emergency services were identified in which the determining factor was an adverse event related to the medication, which would allow better clarifying the relation between the medications used after discharge and the outcome.

The study displayed guiding elements of quality of care, seeking to subsidize decisions to optimize the transition of care and widen the safety of the aged patient.

## CONCLUSION

This study showed, from the perspective of pharmacotherapy, the factors associated with visits of the elderly to emergency departments. For the elderly with heart failure and high complexity pharmacotherapy, there was an association with the visit to the urgency department within 30 days of discharge from the index hospitalization. The diagnosis of *diabetes mellitus* and chronic renal disease were protective factors for the outcome analyzed.
