# Supplementary material for: Emergency department visits of older adults within 30 days of discharge: analysis from the pharmacotherapy perspective
Source: Einstein (Sao Paulo). 2019 Oct 17;18:eAO4871. doi: 10.31744/einstein_journal/2020AO4871 (PMC6896603; doi:10.31744/einstein_journal/2020AO4871)
Supplement: Translation

# Translation: Visitas de idosos a departamentos de emergência em até 30 dias após a alta hospitalar: análise na perspectiva da farmacoterapia

**Authors:** Fabiana Silvestre dos Santos, Bianca Menezes Dias, Adriano Max Moreira Reis

**Affiliations:** 1 Universidade Federal de Minas Gerais, Belo Horizonte, MG, Brasil.

**Keywords:** Serviço hospitalar de emergência, Idoso, Tratamento farmacológico

## Abstract

**Objetivo:**

Analisar, da perspectiva da farmacoterapia, os fatores associados à visita de idosos a departamentos de emergência em até 30 dias após a alta da internação índice.

**Métodos:**

Foi realizado estudo transversal em hospital público geral, com idosos. Visita a departamento de emergência foi definido como a permanência do idoso nesse serviço por até 24 horas. A complexidade da farmacoterapia foi determinada usando o *Medication Regimen Complexity Index.* Os medicamentos potencialmente inapropriados para idosos foram classificados segundo os critérios *American Geriatric Society/Beers* , de 2015. O desfecho investigado foi a frequência de visita a departamento de emergência em 30 dias após a alta hospitalar. Regressão logística multivariada foi realizada para identificar os fatores associados à visita a departamento de emergência.

**Resultados:**

No estudo, foram incluídos 255 idosos; 67 (26,3%) visitaram departamento emergência em 30 dias após a alta hospitalar. Polifarmácia e medicamentos potencialmente inapropriados para idosos não apresentaram associação estatística significante. O diagnóstico de insuficiência cardíaca e o índice da complexidade da farmacoterapia >16,5 apresentaram associação positiva com visita a departamento de emergência (RC=2,3; IC95%: 1,04-4,94; p=0,048; e RC=2,1; IC95%: 1,11-4,02; p=0,011), respectivamente. Ainda, o diagnóstico de *diabetes mellitus* e a doença renal crônica foram fatores de proteção para o desfecho (RC=0,4; IC95%: 0,20-0,73; p=0,004; e RC=0,3; IC95%: 0,13-0,86; p=0,023).

**Conclusão:**

O diagnóstico de insuficiência cardíaca e o índice da complexidade da farmacoterapia >16,5 apresentaram associação positiva com ocorrência de visita a departamento de emergência dentro de 30 dias após a alta.

## INTRODUÇÃO

O aumento do número de idosos na sociedade determina maior utilização de serviços de saúde, incluindo dos departamentos de emergência.^1^ Visita a departamento de emergência é frequentemente um evento sentinela, pois contribui para o declínio do estado de saúde do idoso, sendo associado com risco elevado de resultados negativos, como revisita a departamentos de emergência, hospitalização, institucionalização e declínio funcional.^2^ A multimorbidade, a utilização de polifarmácia, a complexidade da farmacoterapia e a fragilidade estão entre os fatores associados com esses resultados negativos em saúde.^2 , 3^

O retorno a departamentos de emergência após a alta hospitalar frequentemente reflete uma transição de cuidado executada de forma inadequada. Apesar da importância clínica e da relevância para organização dos sistemas de saúde, o padrão de utilização de serviços de emergência após a alta hospitalar é pouco compreendido e investigado, o que contribui para subestimar a extensão da utilização.^4^

Abordagens centradas apenas na readmissão hospitalar fornecem uma visão incompleta do entendimento do cuidado agudo após a alta hospitalar, dificultando identificar os fatores que contribuem para a necessidade do uso subsequente do sistema de saúde, principalmente dos problemas de saúde manejados em serviços de emergência.^4 , 5^

Os fatores relacionados a idade, funcionalidade, polifarmácia, complexidade da farmacoterapia, medicamentos inadequados ao idoso e multimorbidade podem estar associados com retornos a serviços de emergência após a alta hospitalar.^2 , 3^ Além disso, a utilização de serviços de emergência após a alta hospitalar pode ser considerada preditor de que o processo de transição de cuidado está sendo ineficiente. Esse evento pode causar mais superlotação e aumento de custo nos serviços de saúde.^1 , 4 - 6^

## OBJETIVO

Analisar, da perspectiva da farmacoterapia, os fatores associados à visita de idosos a departamentos de emergência em até 30 dias após a alta da internação índice.

## MÉTODOS

### Delineamento do estudo e cenário

Estudo transversal, com idosos, realizado em hospital público da Região Sudeste do Brasil, responsável pelo atendimento a servidores públicos. Adotou-se idade ≥60 anos, para definição de idoso, conforme estabelecido pela Organização Mundial da Saúde para países em desenvolvimento.^7^

### Amostra

Estimou-se a inclusão mínima de 246 idosos no estudo, considerando as seguintes premissas: população infinita, prevalência de 20% de visita de idosos a serviços de urgência após alta hospitalar,^8^ intervalo de confiança de 95% (IC95%), margem de erro de 5% e estatística de teste sobre uma população. O cálculo amostral foi realizado empregando o software *OpenEpi* , versão 2.0 *.*

### Critérios de seleção

Foram incluídos pacientes idosos internados pelas unidades de clínica médica e geriatria, durante o período de abril a novembro de 2017. Os critérios de exclusão foram definidos como pacientes que foram a óbito na internação índice, tiveram internação índice superior a 60 dias, evadiram-se do hospital, retornaram eletivamente ou perdas de contato após a alta. A primeira admissão no período de realização do estudo foi escolhida como a internação índice.^9^

### Considerações éticas

O estudo foi aprovado pelo Comitê de Ética e Pesquisa da Universidade Federal de Minas Gerais, sob parecer 1.952.130 e CAAE: 63612216.7.0000.5149. Todos os pacientes e/ou responsáveis consentiram em participar por meio do Termo de Consentimento Livre e Esclarecido.

### Coleta de dados

Os pacientes internados no hospital por período superior a 24 horas foram convidados para participarem da pesquisa, sendo esta considerada a internação índice. A identificação dos pacientes foi realizada por relatório informatizado do sistema de internação do hospital investigado. Foram realizados: entrevista inicial, coleta de dados no prontuário eletrônico do paciente e dois acompanhamentos telefônicos após a alta (o primeiro para confirmar os medicamentos prescritos na alta hospitalar, e o segundo, para verificar a ocorrência do evento). O primeiro contato telefônico foi realizado em até 72 horas após a alta e o segundo em 30 dias após a alta.

O desfecho foi a procura por departamentos de emergência em até 30 dias após a alta da hospitalização índice, tanto no hospital investigado quanto em outros serviços de saúde. Definiu-se visita a departamentos de emergência como a permanência do idoso inferior a 24 horas, seguindo parâmetros nacionais^10^ e internacionais^11^ que estabelece tempo de observação em serviço de emergência. Foram coletados, por entrevista, dados sociodemográficos de funcionalidade, por pesquisa no prontuário do paciente, sobre caracterizações clínicas e a farmacoterapia do idoso. Os diagnósticos de admissão e reinternações foram classificados de acordo com a Classificação Internacional de Doenças da décima edição (CID 10). As comorbidades foram avaliadas usando-se o índice de comorbidade de Charlson (ICC).^12^ Os medicamentos potencialmente inapropriados para idoso foram descritos segundo os critérios de *American Geriatric Society* /Beers 2015.^13^ A complexidade da farmacoterapia foi calculada utilizando o índice da complexidade da farmacoterapia,^14^ e a versão traduzida para a língua portuguesa do Brasil do *Medication Regimen Complexity Index* (MRCI).^15^ A complexidade da farmacoterapia foi estratificada em complexidade alta sim (valor do índice de complexidade da farmacoterapia − ICF >16,5) ou não (ICF ≤16,5), segundo normatização do MRCI proposta para idosos do Brasil.^16^ A vulnerabilidade foi avaliada pelo instrumento Protocolo de Identificação do Idoso Vulnerável (VES-13) adaptado transculturalmente para o Brasil do *The Vulnerable Elders Survey* .^17^ Os diagnósticos e os medicamentos de risco para reinternação foram identificados usando a definição de Taha et al.^9^

### Análise estatística

Realizou-se análise descritiva com determinação de frequência para as variáveis dicotômicas, e as variáveis numéricas foram descritas como média±desvio padrão (DP) ou mediana (amplitude interquatil – IQR). O teste de Shapiro Wilk foi utilizado para a análise de normalidade. As variáveis numéricas foram dicotomizadas pela mediana. A associação entre as variáveis independentes e visita a departamento de emergência em 30 dias foi realizada de acordo com o teste *t* de Student, Mann-Whitney, teste do χ^2^ e teste exato de Fisher, observando-se as premissas de cada teste. Calculou-se a razão de chance (RC) com intervalo de confiança de 95%. O nível de significância estatística do estudo foi considerado como p<0,05. As variáveis que apresentaram na análise univariada associação com visita a departamento de emergência com p<0,25 foram selecionadas para a regressão logística multivariada. O método *forward stepwise* foi utilizado para a obtenção do modelo final, permanecendo as variáveis com valor p<0,05. A adequação do modelo final foi avaliada pelo teste de Hosmer-Lemeshow considerando adequado se valor p>0,05.

## RESULTADOS

Foram obtidos 300 participantes no estudo, sendo que, destes, 27 (9%) foram a óbito durante a internação, 1 (0,3%) idoso evadiu-se do hospital, 1 (0,3%) idoso foi transferido para outro serviço de saúde e 3 (1%) pacientes foram excluídos devido à internação prolongada. Dos 268 pacientes que receberam alta hospitalar, ocorreram 5 (1,7%) perdas de contato, 1 (0,3%) desistência do estudo e 7 (2,3%) exclusões devido à reinternação eletiva. Desta forma, 255 participantes foram elegíveis para realização das análises, sendo que, destes, 67 idosos (26,3%) necessitaram procurar um departamento de emergência em 30 dias após a alta hospitalar ( Figura 1 ).

**Figura 1:** Fluxograma do estudo

Entre os 67 idosos que visitaram um departamento de emergência 30 dias após a internação índice, 35 (52,2%) permaneceram no departamento por período inferior a 24 horas e 32 (47,8%) foram reinternados. A frequência de visita a departamento de emergência 30 dias após a internação índice foi de 26,3%, a de reinternação foi 12,5% e a de idosos que permaneceram no departamento por período inferior a 24 horas foi 13,7%.

O departamento de emergência do hospital investigado foi procurado por 54 (80,6%) dos 67 idosos que necessitaram de atendimento de urgência ou emergência. As unidades de pronto atendimento e os prontos-socorros de hospitais do Sistema Único de Saúde (SUS) foram procurados por 8 (12,9%) idosos, e 5 (7,5%) buscaram atendimento em serviços privados ou do sistema de saúde suplementar.

As características sociodemográficas, clínicas e farmacoterápicas dos idosos estão descritas na tabela 1 . Em relação ao sexo e à idade, não houve diferença estatisticamente significativa entre os idosos que visitaram o departamento de emergência e os que não visitaram, sendo que o sexo feminino e idade ≥75 anos foram mais frequente nos dois grupos.

**Tabela 1:** Características sociodemográficas, clínicas e farmacoterápicas dos idosos

Características	Visita a serviço de emergência		Total n=255	Valor de p

	Sim n=67	Não n=188		
Sociodemográfico				
Sexo feminino	44 (65,7)	102 (54,3)	146 (57,3)	0,105
Idade	77,0 (13,0)	75,0 (14,0)	75,0 (13,0)	0,470^*^
Clínicas				
ICC	7,0 (6,0)	5,0 (2,0)	5,0 (2,0)	0,359^*^
VES-13	7,0 (6,0)	4,0 (5,0)	5,0 (6,0)	0,06^*^
Diagnóstico de risco para reinternação	52 (77,6)	145 (77,1)	197 (77,3)	0,541
IC	15 (22,4)	23 (12,2)	38 (14,9)	0,045
Câncer	11 (16,4)	18 (9,6)	29 (11,4)	0,130
DPOC	10 (14,9)	24 (12,8)	34 (13,3)	0,655
Pneumonia	15 (22,4)	38 (20,2)	53 (20,8)	0,706
Diabetes	21 (31,3)	89 (47,3)	110 (43,1)	0,023
AVC	7 (10,4)	38 (20,2)	45 (17,6)	0,332
Farmacoterápicas				
Medicamentos prescritos na alta				
AAS + clopidogrel	1 (2,9)	7 (3,7)	8 (3,1)	0,685^†^
Insulina	10 (14,9)	42 (22,3)	52 (20,4)	0,196
Opioide	13 (19,4)	19 (10,1)	32 (12,5)	0,049
Varfarina	6 (1,5)	8 (4,3)	14 (5,5)	0,207^†^
Enoxaparina	-	1 (0,5)	1 (0,4)	0,737^†^
Fondaparinux	-	-	-	-
Digoxina	-	-	-	-
MPII	122 (64,9)	43 (64,2)	165 (64,7)	0,913
Polifarmácia	49 (73,1)	125 (66,5)	174 (68,2)	0,316
Polifarmácia excessiva	31 (16,5)	14 (20,9)	45 (17,6)	0,417
Medicamentos de risco para reinternação	28 (41,8)	65 (34,6)	93 (36,5)	0,292
Número de medicamentos na alta	6,0 (5,0)	6,0 (4,0)	6,0 (4,0)	0,210*
ICF	12,0 (16,5)	16,5 (14,4)	17,0 (14,5)	0,167*

Resultados expressos como n (%) ou mediana (intervalo interquartil). * Teste exato de Fischer;^†^teste de Mann-Whitney. ICC: índice de comorbidade de Charlson; VES-13: Protocolo de Identificação do Idoso Vulnerável; IC: insuficiência cardíaca; DPOC: doença pulmonar obstrutiva crônica; AVC: acidente vascular cerebral; AAS: ácido acetilsalicílico; MPII: medicamentos potencialmente inapropriados para idosos; ICF: índice de complexidade da farmacoterapia.

Quanto às características clínicas, evidenciou-se diferença estatisticamente significativa entre os grupos (p=0,045), com maior frequência de insuficiência cardíaca entre o grupo de idosos que visitou um departamento de emergência. A proporção de idosos com diabetes foi significativamente maior entre aqueles que não buscaram um departamento de emergência. Em relação à funcionalidade, evidenciou-se diferença estatisticamente significante (p=0,038), com maiores valores de VES13 entre o grupo de idosos que foi atendido em um departamento de emergência. Considerando a presença de diagnóstico de risco, não houve diferença entre os idosos; vale ressaltar a alta prevalência de idosos com esta característica na amostra estudada.

Em relação à farmacoterapia, idosos que tiveram alta hospitalar com prescrição de opioide procuraram mais o serviço de urgência que os indivíduos que não receberam prescrição de medicamentos desse grupo terapêutico (p=0,049). A proporção de idosos em uso de medicamentos de risco para internação não apresentou diferença estatisticamente significativa, mas foi mais prevalente entre os idosos que procuraram atendimento de emergência. A proporção de idosos com complexidade da farmacoterapia alta não apresentou diferença estatisticamente significativa entre os grupos, porém foi discretamente mais frequente entre os idosos que internaram.

Na análise univariada, a visita a departamento de emergência apresentou associação com sexo feminino, insuficiência cardíaca, câncer, *diabetes mellitus* , doença renal crônica, asma, fibrilação atrial, VES-13, complexidade da farmacoterapia alta, uso de insulina, opioide e varfarina. No modelo multivariado final permaneceram as seguintes variáveis: insuficiência cardíaca (RC= 2,3, IC95%: 1,04-4,94), *diabetes melitus* (RC=0,4; IC95%: 0,20-0,73), doença renal crônica (RC=0,3; IC95%: 0,13-0,9), complexidade da farmacoterapia alta (RC=2,1; IC95%: 1,11-4,02) como apresentado na tabela 2 .

**Tabela 2:** Análise univariada e multivariada dos fatores associados à visita de idosos a departamento emergência em 30 dias após a alta hospitalar

Variável	Frequência de ocorrência de reintenação		Análise univariada		Análise multivariada		
		
	Sim	Não	Razão de chance (IC95%)	Valor de p	Razão de chance (IC95%)		Valor de p*
Sociodemográficas							
Sexo							
Feminino	44 (65,7)	102 (54,3)	1,6 (0,90-2,88)	0,105	---		---
Masculino	23 (34,3)	86 (45,7)	1				
Idade (anos)							
≥75	39 (58,2)	101 (53,7)	1,2 (0,68-2,19)	0,526	---		---
<75	28 (41,8)	87 (46,3)	1				
Clínicas							
Dias de internação							
≥12	40 (59,7)	101 (53,7)	1,3 (0,72-2,25)	0,398	---		---
<12	27 (40,3)	87 (46,3)	1				
Doenças (CID10)							
Acidente vascular encefálico (I64)							
Sim	11 (16,4)	26 (13,8)	1,2 (0,57-2,64)	0,606	---		---
Não	56 (83,6)	162 (86,2)	1				
Insuficiência cardíaca (I50)							
Sim	15 (22,4)	23 (12,2)	2,1 (1,01-4,26)	0,045	2,3 (1,04-4,94)		0,048
Não	52 (77,6)	165 (87,8)	1		1		
DPOC (J44)							
Sim	10 (14,9)	24 (12,8)	1,2 (0,54-2,66)	0,655	---		---
Não	57 (85,1)	164 (87,2)	1				
Câncer (C00-C75)							
Sim	11 (16,4)	18 (9,6)	1,9 (0,83-4,16)	0,130	---		---
Não	56 (83,6)	170 (90,4)	1				
*Diabetes mellitus* (E10-E14)							
Sim	21 (31,3)	89 (47,3)	0,5 (0,28-0,92)	0,023	0,4 (0,20-0,73)		0,004
Não	46 (68,7)	99 (52,7)	1		1		
Pneumonia (J12-J18)							
Sim	15 (22,4)	38 (20,2)	1,1 (0,58-2,24)	0,706	---		---
Não	52 (77,6)	150 (79,8)	1				
Demência (F00-F03)							
Sim	22 (32,8)	57 (30,3)	1,1 (0,62-2,05)	0,702	---		---
Não	45 (67,2)	131 (69,7)	1				
Doença renal crônica (N18)							
Sim	7 (10,4)	38 (20,2)	0,5 (0,20-1,09)	0,072	0,3 (0,13-0,86)		0,023
Não	60 (89,6)	150 (79,8)	1		1		
Asma (J45)							
Sim	2 (3,0)	1 (0,5)	5,8 (0,51-64,51)	0,170^*^	---		---
Não	65 (97,0)	187 (99,5)	1				
Fibrilação atrial (I48)							
Sim	3 (4,5)	18 (9,6)	0,4 (0,13-1,55)	0,193	---		---
Não	64 (95,5)	170 (90,4)	1				
Doença hipertensiva (I10-I15)							
Sim	50 (74,6)	131 (69,7)	1,3 (0,68-2,41)	0,444	---		---
Não	17 (25,4)	57 (30,3)	1				
Cuidados paliativos							
Sim	8 (11,9)	14 (7,4)	1,7 (0,67-4,22)	0,261	---		---
Não	59 (88,1)	174 (92,6)	1				
Índice de comorbidade de Charlson							
≥5	39 (58,2)	104 (55,3)	1,1 (0,64-1,98)	0,682	---		---
<5	28 (41,8)	84 (44,7)	1				
Funcionalidade							
VES-13							
≥5	43 (64,2)	93 (49,5)	1,8 (1,03-3,26)	0,038	---		---
<5	24 (35,8)	95 (50,5)	1				
Farmacoterápicas							
AAS + clopidogrel							
Sim	1 (1,5)	7 (3,7)	0,4 (0,05-3,25)	0,685^*^	---		---
Não	66 (98,5)	181 (96,3)	1				
Insulina							
Sim	10 (14,9)	42 (22,3)	0,6 (0,29-1,30)	0,196	---		---
Não	57 (85,1)	146 (77,7)	1				
Opioide							
Sim	13 (19,4)	19 (10,1)	2,1 (0,99-4,62)	0,049	---		---
Não	54 (80,6)	169 (89,9)	1				
Varfarina							
Sim	6 (9,0)	8 (4,3)	2,2 (0,74-6,63)	0,207^*^	---		---
Não	61 (91,0)	180 (95,7)	1				
MPII							
Sim	43 (64,2)	122 (64,9)	0,7 (0,54-1,74)	0,916	---		---
Não	24 (35,8)	66 (35,1)	1				
Polifarmácia							
Sim	49 (73,10	125 (66,5)	1,4 (0,74-2,55)	0,316	---		---
Não	18 (26,9)	63 (33,5)	1				
MRCI alto							
Sim	40 (59,7)	93 (49,5)	1,5 (0,86-2,67)	0,150	2,1 (1,11-4,02)		0,021
Não	27 (40,3)	95 (50,5)	1		1		

Resultados expressos como n (%). Teste de Hosmer-Lemeshow: χ^2^=1,549. Grau de Liberdade = 5; p=0,907. * Valores de p com variáveis calculadas pelo teste exato de Fisher. CID10: Classificação Internacional de Doenças, décima edição; DPOC: doença pulmonar obstrutiva crônica; VES-13: Protocolo de Identificação do Idoso Vulnerável; AAS: ácido acetilsalicílico; MPII: medicamentos potencialmente inapropriados para idoso; MRCI: *Medication Regimen Complexity Index* .

## DISCUSSÃO

No presente estudo, cerca de um quarto dos idosos retornou a departamentos de emergência em 30 dias após a alta hospitalar, e essa visita apresentou uma associação positiva com insuficiência cardíaca e complexidade da farmacoterapia, enquanto que *diabetes mellitus* e doença renal crônica foram fatores de proteção para o desfecho. O retorno não planejado a departamentos de emergência em 30 dias após a internação é pouco pesquisado, e os estudos desenvolvidos até o momento alertam para a importância de ampliar as investigações sobre esse período, para incrementar o conhecimento sobre o cuidado agudo baseado em hospitais de pacientes com alta hospitalar recente.^4 , 5 , 8 , 18^ A análise do cuidado prestado durante a internação considerando somente a readmissão hospitalar não propicia uma visão integral do cuidado agudo e limita o desenvolvimento de ações em prol da melhoria da transição do cuidado, especialmente dos pacientes idosos.

As taxas de visita a departamentos de emergência variaram de 7,5 a 23,8 em estudos prévios,^4 , 5 , 8 , 18^ porém essas investigações não abrangeram exclusivamente idosos e incluíram pacientes cirúrgicos. Não foram identificados estudos desenvolvidos no Brasil. A frequência de visita a departamento de emergência (26,3%) aproxima-se dos estudos com maior frequência. Entretanto, a comparação entre estudos é dificultada, devido às variações metodológicas, à falta de uniformidade nos prazos que caracterizam visita a departamentos de emergência e à exclusão em alguns estudos dos pacientes que visitam o serviço de emergência e internam, assim como pelas diferenças na organização do cuidado em emergência nos países onde os estudos foram desenvolvidos.

O retorno a serviços de emergência relacionados à insuficiência cardíaca é um importante desfecho negativo, resultando também em importantes custos de cuidados em saúde. A taxa de retorno a serviços de emergência por pacientes com insuficiência cardíaca foi de 20% em estudo canadense.^19^ Em outra investigação, pacientes com esse diagnóstico apresentando idade ≥80 anos eram significativamente mais propensos ao retorno, se comparados àqueles com idade inferior.^20^ Ainda, em ambos os estudos, o uso de medicamentos antiarrítmicos esteve associado negativamente à ocorrência do desfecho.^19 , 20^

A farmacoterapia de pacientes com insuficiência cardíaca é complexa e abrange medicamentos de várias classes terapêuticas. Além disso, as múltiplas comorbidades que os idosos apresentam aumentam ainda mais a complexidade do regime medicamentoso. Regimes medicamentosos complexos estão associados com má adesão ao tratamento,^21 , 22^ polifarmácia^21 , 23^ e hospitalização por eventos adversos.^24^ A descompensação aguda da insuficiência cardíaca é um importante determinante da busca por departamentos de emergência.^25 , 26^ A não adesão devido à complexidade da farmacoterapia ou o uso incorreto de medicamentos podem contribuir para a descompensação aguda da insuficiência cardíaca.^27^ Escores elevados do MRCI na alta hospitalar fornecem avaliação mais global do risco associado com o regime medicamentoso complexo. Pacientes com valores mais elevados de MRCI apresentam maior probabilidade de adoecer e apresentar mais resultados negativos.^21 , 24^ Esses fatores podem explicar a associação positiva entre visita a departamentos de emergência, insuficiência cardíaca e complexidade da farmacoterapia elevada.

A ausência de associação entre complexidade da farmacoterapia >15 (dicotomizada pela média) e visita a departamento de emergência em 30 dias foi descrita para indivíduos com idade maior de 50 anos.^3^ A divergência em relação a associação detectada no nosso estudo pode ser atribuída ao perfil diferente da população investigada, pois os idosos apresentam farmacoterapia mais complexa. Além disso, foi adotada a definição de complexidade elevada (complexidade da farmacoterapia >16,5) normatizada para idosos do Brasil,^16^ que permite classificação mais adequada.

A intensificação da farmacoterapia para o tratamento do *diabetes mellitus* (mensurada pelo aumento de dose de medicamentos hipoglicemiantes, adição de outros medicamentos hipoglicemiantes e adição de insulina) diminuiu significativamente o risco de retorno em 30 dias ao setor de urgência após a alta em um hospital norte-americano.^28^ É possível inferir que, entre os idosos estudados, ocorreu intensificação da farmacoterapia, que contribuiu para o controle adequado da doença no período de 30 dias, explicando a associação inversa identificada neste estudo entre ter *diabetes mellitus* e visita a departamento de emergência em 30 dias após a alta. Entretanto, vale destacar que insulina, usada por alguns pacientes com diabetes, é um medicamento de risco, principalmente para idosos, o que pode contribuir para a busca por serviços de saúde após a alta hospitalar.^9^ No entanto, na ótica da farmacoterapia, a menor procura por departamentos de emergência em 30 dias pelos idosos com doença renal crônica pode também ser atribuída à otimização do tratamento.

Investigação sobre o perfil e a prevalência de procura de serviços de saúde por indivíduos com diabetes mostrou menor prevalência em pacientes com tempo mais longo de diagnóstico da doença, como os idosos.^29^ Os idosos com diabetes podem apresentar melhor autocuidado em função do maior conhecimento da doença e do tratamento, em virtude do diagnóstico realizado há vários anos, explicando o fator de proteção encontrado para busca por serviços de emergência. O melhor cuidado para saúde e, especialmente, pelas mulheres explica a associação com doença renal crônica. Mulheres com doença renal crônica apresentam menor taxa de reinternação hospitalar em 30 dias. A maior frequência de mulheres na casuística investigada pode justificar a associação. Porém, vale destacar que medicamentos constituem importante determinante de internação hospitalar de indivíduos com doença renal crônica.^6^

A ausência de associação independente entre polifarmácia e retorno ao departamento de emergência foi descrita por uma coorte de idosos italianos,^2^ o que está em consonância com os achados do presente estudo. Entretanto, para os idosos da mesma coorte, foi encontrada associação independente entre polifarmácia excessiva (uso de dez ou mais medicamentos, considerando os últimos 3 meses) e visita ao departamento de emergência 30 dias após a alta.^2^ Os autores ressaltam que polifarmácia é marcador de multimorbidade, gravidade de doença, fragilidade e de síndromes geriátrica,^2^ condições que podem contribuir para resultados negativos em saúde. É importante considerar polifarmácia como um parâmetro para orientar ações na transição do cuidado, visando evitar eventos adversos no idoso no futuro, pois, com 6 meses de seguimento, tanto polifarmácia como polifarmácia excessiva apresentaram associação independente com retorno ao departamento de emergência.^2^

Nosso estudo apresentou informações importantes para orientar ações visando otimizar a transição do cuidado de idosos e reduzir visitas ao departamento de emergência. Além disso, é importante destacar que se trata de investigação prospectiva, na qual foram feitos acompanhamentos telefônicos após a alta, garantindo dados fidedignos sobre o desfecho. Outra força do estudo é a determinação da associação entre visita a departamento de emergência e complexidade da farmacoterapia utilizando definição exata de complexidade elevada (MRCI >16,5), estabelecida na normatização do MRCI para idosos brasileiros.^16^

No entanto, apresentamos algumas limitações, como sua realização em um único hospital que atendia exclusivamente servidores públicos, o que limita generalizações para pacientes atendidos em hospitais do SUS ou da rede particular. Segundo, não foram incluídos, no estudo, idosos provenientes de clínicas cirúrgicas, o que pode ter subestimado a frequência de busca de departamento de emergência. Por fim, não foram identificadas as visitas ao serviço de emergência que tiveram como determinante um evento adverso relacionado a medicamento, o que permitiria elucidar melhorar a relação entre os medicamentos utilizados após alta e o desfecho.

O estudo apresentou elementos orientadores da qualidade do cuidado, visando subsidiar decisões para otimizar a transição do cuidado e ampliar a segurança do paciente idoso.

## CONCLUSÃO

O estudo mostrou, da perspectiva da farmacoterapia, os fatores associados à visita de idosos a departamento de emergência. Para os idosos com insuficiência cardíaca e complexidade de farmacoterapia alta, houve associação positiva com a ocorrência de visita a departamento de urgência em até 30 dias após a alta da internação índice. O diagnóstico de *diabetes mellitus* e a doença renal crônica foram fatores de proteção para o desfecho analisado.
